# Prevalence and correlates of diagnosed and undiagnosed hypertension in the indigenous Kuna population of Panamá

**DOI:** 10.1186/s12889-019-7211-5

**Published:** 2019-06-28

**Authors:** Daniel R. Hanna, Rebekah J. Walker, Brittany L. Smalls, Jennifer A. Campbell, Aprill Z. Dawson, Leonard E. Egede

**Affiliations:** 1 College of Medicine, Medical College of Wisconsin, Milwaukee, WI USA; 2 Division of General Internal Medicine, Department of Medicine, Medical College of Wisconsin, 8701 Watertown Plank Rd, Milwaukee, WI 53226-3596 USA; 3 Center for Advancing Population Science, Medical College of Wisconsin, Milwaukee, WI USA; 4 Center for Health Services Research, Department of Internal Medicine, University of Kentucky, Lexington, KY USA

**Keywords:** Hypertension, Indigenous, Latin America, Income, Undiagnosed hypertension

## Abstract

**Background:**

To determine the prevalence of hypertension and investigate sociodemographic correlates in an indigenous Kuna community living on the San Blas islands of Panama.

**Methods:**

Data was collected from adults using a paper-based survey using a cross sectional study design. Blood pressure was measured, and hypertension defined at two cut-points: 130/80 mmHg and 140/90 mmHg. Individuals with undiagnosed hypertension had a blood pressure measurement that indicated hypertension, however, the individual had not been told by a doctor they had hypertension. Whereas individuals with diagnosed hypertension had been told by a healthcare provider that they had hypertension. Univariate tests compared diagnosed and undiagnosed hypertension by sociodemographic categories and logistic regression models tested individual correlates adjusting for all sociodemographic factors.

**Results:**

Two hundred and eleven adult indigenous Kuna participated in the study. Overall prevalence of hypertension was 6.2% (95%CI:3.32–10.30) as defined by 140/90 mmHg, and 16.6% (95%CI:11.83–22.31) as defined by 130/80 mmHg. Hypertension was significantly higher in men (31.6, 95% CI:19.90–45.24, compared to 11.0, 95% CI:6.56–17.09). Individuals with low income were 3 times more likely to be hypertensive (OR = 3.13, 95% CI:1.02–9.60) and 3.5 times more likely to have undiagnosed hypertension (OR = 3.42, 95% CI:1.01–11.52); while those with moderate income were 6 times more likely to be hypertensive (OR = 7.37, 95% CI:1.76–30.90) compared to those who were poor.

**Conclusion:**

The prevalence of diagnosed and undiagnosed hypertension is higher in men and those with higher income. Investigating these factors remains vitally important in helping improve the health of the Kuna through targeted interventions to address chronic disease.

## Background

Hypertension is a significant health problem, affecting 31.1% of the global population, and leads to serious morbidity and mortality [1]. Sustained elevated blood pressure is linked to the adverse health outcomes of cardiovascular disease, stroke, and chronic renal disease [2]. While mortality due to cardiovascular disease has declined recently, it remains the leading cause of death in Western Europe, North America, and Latin America [3, 4]. In addition, given its prevalence and health consequences, hypertension has been deemed the most meaningful metabolic risk factor globally and is the leading contributor to preventable deaths [5]. Hypertension was previously defined as a blood pressure with a systolic pressure of greater than 140 mmHg and or a diastolic pressure greater than 90 mmHg, however an update in the 2017 High Blood Pressure Clinical Practice Guidelines recommends hypertension be defined as blood pressure with a systolic reading of 130 mmHg or greater and a diastolic reading of 80 mmHg or greater [6, 7]. This change in guidelines, and health effects linked to socioeconomic transitions in countries globally, highlights the importance of understanding the prevalence and drivers of hypertension in communities [8].

There are approximately 400 million indigenous people in the world today, and they experience a disproportionate level of poverty and poor health outcomes in comparison to the general population [9–11]. For example, indigenous populations experience higher levels of infant and maternal mortality, malnutrition, and infectious disease burden [9–12]. Changes in traditional lifestyles and diets have also influenced indigenous health, resulting in increasing incidences of chronic disease including type 2 diabetes, obesity, and hypertension [9, 10]. In a review of hypertension among indigenous populations of North America, rates of hypertension were found to be 23.5%, and the Fulani population of Cameroon were found to have a prevalence of 31.1% [13, 14]. The high prevalence in the Fulani was suggested to be a result of acculturation and transition away from traditional diet to a more westernized diet [14]. Other studies suggest sociodemographic factors, such as education, are associated with higher rates of hypertension in indigenous populations [15]. However, lack of access to healthcare is also a common theme in indigenous research, which can result in additional burden due to undiagnosed hypertension [9–11]. This concern is especially prevalent in the indigenous populations of Latin America and the Caribbean, where many indigenous groups live in remote or inaccessible areas [11, 12, 16]. Indigenous populations in Latin America have also been noted for a large poverty gap, with the probability of poverty increasing over the past decade [11]. Finally, the highly variable nature of social, environmental, and cultural factors present in indigenous communities makes generalization between groups difficult and suggest the importance of understanding unique characteristics in each group [9, 10, 17].

The Kuna Indians are an indigenous population native to the islands off the eastern coast of Panamá [18]. They have been of particular interest in previous studies due to the seeming lack of hypertension among the island-dwelling Kuna, but increased prevalence among the Kuna who lived on the mainland [19]. This finding prompted a number of studies into why this population saw little to no age-related increase in blood pressure [19–24]. Diet was the focus of the possible explanation, in particular the increased intake of flavanol rich cocoa by the island dwelling Kuna [20, 23]. A recent study investigating the prevalence of hypertension in Panamá concluded that the overall prevalence of hypertension was 29.6% [25]. They found that prevalence of high blood pressure was lower in the Kuna Indians, at 11.4%, but those included in the study lived in indigenous zones on the mainland rather than the more remote San Blas island communities [25].

It has been nearly two decades since data was collected in the more remote Kuna populations residing in the San Blas islands regarding prevalence of chronic disease and drivers of hypertension. Therefore, the objective of this study was to determine the prevalence of hypertension and investigate sociodemographic correlates in an indigenous Kuna community living on the San Blas islands separated from the mainland.

## Methods

### Research approvals

The research project was developed by Indigenous Health International (IHI), an approved 501(c) [3] non-profit organization, in collaboration with the Panamá Ministry of Health and the Kuna Congress. Development of the research program and efforts to deliver care and conduct needs assessments regarding health of indigenous communities in Panamá by IHI has been described elsewhere [26]. Research activities were approved by the Director of Indigenous Health in Panamá, the national Kuna Congress, and local Kuna community leadership.

The current research protocol, research procedures, and study related documentation were also approved by the Western Institutional Review Board (WIRB). The WIRB, is an accredited organization that has been providing human subjects and regulatory compliance for more than 40 years across 70 countries. As approved by the WIRB, participants provided verbal consent process in lieu of written consent, verbalizing that they were aware that they were taking part in a research study and understood the risks and benefits associated with participation. Verbal consent was approved by the WIRB as a more culturally appropriate method of consenting given literacy of the population and lack of experience with written consent within the culture.

### Data collection

**Fig. 1:**
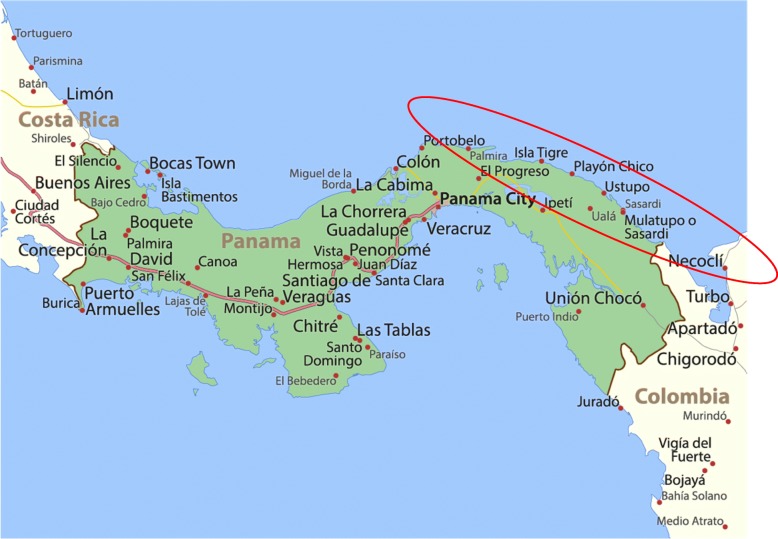
Location of San Blas Indigenous Communities in Panama This image is licensed under ID #192515131, License: Standard

This study used a cross-sectional study design. Recruitment took place at the local clinic and throughout the larger Ustupu and Ogobsucum Kuna communities over a one-week period in 2013 using a convenience sample and a consecutive sampling design. The communities are located off the eastern coast of Panama and have limited access to healthcare. (see Fig. 1) Recruitment methods are also described elsewhere and summarized here [26]. Possible participants were approached to determine interest in study participation. In addition, local leadership announced the study during community meetings. Inclusion criteria were 1) Age 18 or older, or age of majority; 2) residence in administrative region of indigenous community. Interested participants were provided an overview of the study and were asked if they were still interested in participating. Participants were given the option of filling out a paper-based survey independently or having the paper-based survey read to them. As regular census data for the community does not exist, recruitment was not based on a sampling frame. The research team approached 230 individuals, among which 19 (8%) declined to participate. Each survey was checked for missing data and any missing data points were obtained by participants prior to completion of the survey.

Data was collected using paper-based surveys. Questions were developed using standardized health outcomes, measures from the “Panamanian prevalence of risk factors associated with cardiovascular disease in 18 year or older population survey”, a national survey used in Panamá to assess CVD risk factors in an adult population in 2010 [27], validated measures for psychosocial factors, and standardized health outcome measures. Each survey was checked for missing data and any missing data points were obtained by participants prior to completion of the survey.

### Outcomes

#### Blood pressure measurement

Blood pressure was taken at the time of the survey and was measured using the automated OMRON BP742N blood pressure monitor. Participants were seated with feet flat on the floor for at least 5 min prior to obtaining their blood pressure reading and the reading was an average of 3 measurements, with a one-minute interval between readings taken by the automated machine. The blood pressure cuff was placed on the left arm of each participant, unless a request was made to use the right arm, or the left arm was injured. Blood pressure was collected by team members after training and observation of collection by a board-certified physician. All blood pressure measurements were observed by at least one, and generally two study members.

#### Hypertension diagnosis

Hypertension was determined based on two clinically accepted cut-points: > = 130/80 mmHg and > =140/90 mmHg [28]. Blood pressure measurement averages were used to dichotomize each individual as diagnosed based on each cut-point.

#### Hypertension awareness

Participants were asked “Has any doctor or healthcare worker ever told you that you have high blood pressure or hypertension?” and responded either “yes” or “no”. Individuals who responded yes to either doctor or healthcare provider were categorized as having high blood pressure.

#### Undiagnosed hypertension

Individuals who responded they had not been told by a doctor or healthcare professional they had hypertension, but who would be diagnosed with hypertension based on the > = 130/80 mmHg cut point were considered to have undiagnosed hypertension.

### Demographic variables

Standard demographic variables were collected using self-reported questions on age (as a continuous variable); sex (as a dichotomous male/female); education was categorized into groups--- primary (0–6 years of school), secondary (7–12 years of school), some college (13–17), and college graduate or higher (18+); and monthly family income. Monthly income was collected as annual estimates are less valid in international samples where annual tax returns are not completed. As no published data exists regarding expected levels of income we relied on community input for interpretation. Based on conversation with community leaders to understand the meaning of income levels in the context of the community, monthly income was categorized as poor (report of unknown levels of income indicating subsistence farming/fishing), low income (<$250 balboa per month), and moderate income (greater than $250 balboa per month). For reference to US dollars, $1 balboa equals $1 US. The number of individuals supported by the family income was also asked. Marital status was asked with single, separated, divorced, or widowed categorized as ‘not married’ and married or other union categorized as ‘married’. Literacy was assessed by asking whether respondents were able to read and write.

### Statistical analysis

First, mean and standard deviation were used to describe continuous variables and frequencies were used to describe categorical variables for demographic factors in the sample (age, sex, education, monthly income, marital status, number of dependents, and literacy). Secondly, demographic information was investigated in relation to each of the three outcomes using chi2 tests, proportions, and confidence intervals (> = 130/80 mmHg cut-point, > = 140/90 mmHg cut-point and undiagnosed hypertension). Third, we performed two multiple variable logistic regression models to understand the independent correlates of hypertension and undiagnosed hypertension. In the first model, hypertension at the cut-point of > = 130/80 served as the outcome with demographic factors serving as independent variables. In the second model, undiagnosed hypertension served as the outcome with demographic factors serving as independent variables. It was determined a priori to keep all variables regardless of significance in the model to allow investigation of independent correlates accounting for other factors. All analysis was run using Stata v.14. Significance was determined based on a two tailed alpha of *p* < 0.05.

## Results

A combination of direct contact and snowball sampling methods were used, yielding a response rate of 92%. Table 1 describes the 211 adults participating in the study. A higher proportion of the sample was female at 73% compared to 27% males. The mean age was 47.6 (standard deviation: 18.7) years, and age was spread with 40.1% between 18 and 39 years, 32.4% between 40 and 59 years, and 27.5% between 60 and 90 years. The largest portion of the population (53.8%) had secondary/university level education, yet about one-fifth (20.7%) indicated they did not know how to read or write. 51.9% of participants were categorized as poor based on monthly income while 37% made less than $250 (low income) and 11.1% reporting at least $250 in monthly income (moderate income).

**Table 1:** Sample Demographics for Adult Kuna Participants in 2012

*n* = 211	n (%)
Sex	
Male	57 (27.0)
Female	154 (73.0)
Age in years	
Mean (SD)	47.6 (18.7)
18–39	83 (40.1)
40–59	67 (32.4)
60–90	57 (27.5)
Education	
None	35 (17.6)
Primary	57 (28.6)
Secondary/University	107 (53.8)
Monthly Income	
Poor	108 (51.9)
Low Income	77 (37.0)
Moderate Income	23 (11.1)
Married	
No	56 (29.6)
Yes	133 (70.4)
Literacy	
No	43 (20.7)
Yes	165 (79.3)
Family Dependents	
Median	8
1 to 5	52 (25.0)
6 to 8	61 (29.3)
9 to 11	50 (24.0)
12 to 22	45 (21.6)

*SD* Standard Deviation

Table 2 provides the prevalence of hypertension characterized as blood pressure above two cut-points: > = 140/90 mmHg and > =130/80 mmHg. Six point 2 %(6.2%,*n* = 13) (95% CI: 3.32–10.30) of the population had high blood pressure as defined by > = 140/90 mmHg, and 16.6% (*n* = 35) (95% CI: 11.83–22.31) had high blood pressure as defined by > = 130/80 mmHg. There were no significant differences by sex, age, or education in the prevalence of > = 140/90 mmHg hypertension. However, there were significant differences by monthly income, with 26.1% (*n* = 6) (95% CI: 10.23–48.40) in the moderate income category, 5.2% (*n* = 4) (95% CI: 1.43–12.77) in the low income category, and 2.8% (n = 3) (95% CI: 0.58–7.90) in the poor category. For > = 130/80 mmHg hypertension, sex and age were significantly different (Table 2). Finally, similar to > = 140/90 mmHg, the cut-point of > = 130/80 mmHg showed differences in hypertension by income (Table 2).

**Table 2:** Prevalence of Hypertension in Kuna Population Sampled using Two Cut-points

Hypertension above 140/90 mmHg				Hypertension above 130/80 mmHg			
	n (%)	95% CI	*P*-value		n (%)	95% CI	*P*-value
Overall	13 (6.2)	3.32–10.30		Overall	35 (16.6)	11.83–22.31	
Sex			0.11	Sex			< 0.001
Male	6 (10.5)	3.96–21.51		Male	18 (31.6)***	19.90–45.24	
Female	7 (4.5)	1.85–9.14		Female	17 (11.0)***	6.56–17.09	
Age			0.22	Age			0.033
18–39	5 (6.0)	1.98–13.50		18–39	7 (8.4)*	3.46–16.61	
40–59	2 (3.0)	0.36–10.37		40–59	12 (17.9)*	9.61–29.20	
60–90	6 (10.5)	3.96–21.52		60–90	14 (24.6)*	14.13–37.76	
Education			0.59	Education			0.89
None	2 (5.7)	0.70–19.16		None	5 (14.3)	4.81–30.26	
Primary	2 (3.5)	0.43–12.11		Primary	10 (17.5)	8.75–29.91	
Secondary/University	8 (7.5)	3.28–14.20		Secondary/University	19 (17.7)	11.04–26.33	
Monthly Income			**< 0.001**	Monthly Income			**0.001**
Poor	3 (2.8)***	0.58–7.90		Poor	9 (8.3)***	3.88–15.23	
Low Income	4 (5.2)***	1.43–12.77		Low Income	18 (23.4)***	14.48–34.40	
Moderate Income	6 (26.1)***	10.23–48.40		Moderate Income	8 (34.8)***	16.38–57.26	

**Bold** indicates significance at *p* = 0.05–0.1 *p < 0.05 **p < 0.01 ***p < 0.001 based on chi2 test

*CI* confidence interval

Table 3 presents the prevalence of hypertension that was not previously recognized as compared to population demographics. Men were significantly more likely to have undiagnosed hypertension at 25% (*n* = 14) (95% CI: 14.39–38.37) and individuals with higher levels of income were significantly more likely to have undiagnosed hypertension with 22.7% (*n* = 5) (95% CI: 7.82–45.37) of moderate income and 18.9% (n = 14) (95% CI: 10.75–29.70) of low income compared to poor.

**Table 3:** Prevalence of Undiagnosed Hypertension in Adult Kuna Population Sampled

	n (%)	95% CI	*P*-value
Sex			**0.002**
Male	14 (25.0)**	14.39–38.37	
Female	13 (8.8)**	4.79–14.65	
Age			0.15
18–39	6 (7.3)	2.73–15.25	
40–59	9 (14.7)	6.97–26.17	
60–90	10 (17.8)	8.91–30.40	
Education			0.75
None	5 (15.6)	5.27–32.79	
Primary	6 (10.7)	4.03–21.87	
Secondary/University	15 (14.6)	8.39–22.88	
Monthly Income			**0.04**
Poor	8 (7.7)*	3.38–14.59	
Low Income	14 (18.9)*	10.75–29.70	
Moderate Income	5 (22.7)*	7.82–45.37	

**Bold** indicates significance at *p* = 0.05–0.1 *p < 0.05 **p < 0.01 ***p < 0.001 based on logistic regression

*CI* confidence interval

Table 4 presents the regression model for the independent correlates of hypertension. Sex, education, and marital status were not significantly associated with hypertension after adjusting for all demographic factors. Those aged 40–59 years were 2.8 times more likely than those aged 18–39 years to have hypertension (OR = 2.77, 95% CI: 0.83–9.22). In addition, individuals with low income were 3.1 times more likely to be hypertensive (OR = 3.13, 95% CI: 1.02–9.60), and those with moderate income were 7.3 times more likely (OR = 7.37, 95% CI: 1.76–30.90) to have hypertension compared to those who were poor.

**Table 4:** Independent Correlates of Hypertension (130/80 mmHg) in Adult Kuna Population Sampled

	N	n (%)	Univariable Analysis		Multivariable Analysis	
			Odds Ratio (95% CI)	*P*-value	Odds Ratio (95% CI)	*P*-value
Sex						
Male (Ref)	57	18 (31.6%)	Ref		Ref	
Female	154	17 (11.0%)	0.27** (0.13–0.57)	**0.001**	0.50 (0.17–1.52)	0.23
Age						
18–39 (Ref)	83	7 (8.4%)	Ref		Ref	
40–59	67	12 (17.9%)	2.37 (0.88–6.40)	0.089	2.77 (0.83–9.22)	0.096
60–90	57	14 (24.6%)	3.53* (1.32–9.43)	**0.012**	3.46 (0.77–15.51)	0.10
Education						
None (Ref)	35	5 (14.3%)	Ref		Ref	
Primary	57	10 (17.5%)	1.28 (0.40–4.10)	0.68	0.83 (0.16–4.24)	0.82
Secondary/University	107	19 (17.8%)	1.30 (0.44–3.77)	0.64	1.30 (0.22–7.65)	0.77
Monthly Income						
Poor (Ref)	108	9 (8.3%)	Ref		Ref	
Low Income	77	18 (23.4%)	3.36** (1.42–7.95)	**0.006**	3.13* (1.02–9.60)	**0.046**
Moderate Income	23	8 (34.8%)	5.87** (1.96–17.56)	**0.002**	7.37** (1.76–30.90)	**0.006**
Marital Status						
No (Ref)	56	7 (12.5%)	Ref		Ref	
Yes	133	21 (15.8%)	1.31 (0.52–3.29)	0.56	1.13 (0.38–3.31)	0.83

**Bold** indicates significance at *p* = 0.05–0.1 *p < 0.05 **p < 0.01 ***p < 0.001 based on logistic regression

Model is adjusted for sex, age, education, monthly income, and marital status

*CI* confidence interval

Table 5 presents the regression model for the independent correlates of undiagnosed hypertension. Age, education, and marital status were not significantly associated with undiagnosed hypertension after adjusting for all demographic factors. An association with sex showed a trend toward significance, with females being 65% less likely than males to have undiagnosed hypertension (OR = 0.35, 95% CI: 0.11–1.15). Monthly income once again showed a significant association when comparing groups to poor individuals. Those with low income were 2.42 times more likely to have undiagnosed hypertension (OR = 3.42, 95% CI: 1.01–11.52) compared to poor.

**Table 5:** Independent Correlates of Undiagnosed Hypertension in Adult Kuna Population Sampled

	N	n (%)	Univariable Analysis		Multivariable Analysis	
			Odds Ratio (95% CI)	*P*-value	Odds Ratio (95% CI)	*P*-value
Sex						
Male (Ref)	56	14 (25%)	Ref		Ref	
Female	147	13 (8.8%)	0.29** (0.13–0.67)	**0.004**	0.35 (0.11–1.15)	0.083
Age						
18–39 (Ref)	82	6 (7.3%)	Ref		Ref	
40–59	61	9 (14.8%)	2.19 (0.74–6.53)	0.16	1.88 (0.53–6.72)	0.33
60–90	56	10 (179%)	2.75 (0.94–8.08)	0.065	2.22 (0.47–10.53)	0.31
Education						
None (Ref)	32	5 (15.6%)	Ref		Ref	
Primary	56	6 (10.7%)	0.65 (0.18–2.32)	0.51	0.40 (0.07–2.36)	0.32
Secondary/University	103	15 (14.6%)	0.92 (0.31–2.77)	0.88	0.71 (0.10–4.75)	0.72
Monthly Income						
Poor (Ref)	104	8 (7.7%)	Ref		Ref	
Low Income	74	14 (18.9%)	2.80* (1.11–7.07)	**0.029**	3.42* (1.01–11.52)	**0.047**
Moderate Income	22	5 (22.7%)	3.53* (1.03–12.08)	**0.045**	3.96 (0.82–19.20)	0.087
Marital Status						
No (Ref)	53	6 (11.3%)	Ref		Ref	
Yes	128	18 (14.1%)	1.28 (0.48–3.43)	0.62	1.14 (0.36–3.61)	0.83

**Bold** indicates significance at *p* = 0.05–0.1 *p < 0.05 **p < 0.01 ***p < 0.001 based on logistic regression

Model is adjusted for sex, age, education, monthly income, and marital status

*CI* confidence interval

## Discussion

Among the sample of Kuna Indians living on the San Blas islands off the coast of Panamá, we found an overall prevalence of hypertension characterized by > = 130/80 mmHg to be 16.6%. The prevalence was significantly higher among males as compared to females, and 25% of the men were unaware of their condition as compared to only 8% of the women. Increasing age was correlated with increased prevalence, which is typical of previous studies of hypertension, however, it was not as strongly associated as sex. Of particular interest was the gradient relationship found between income and hypertension. Individuals with low income were 3 times more likely to have diagnosed hypertension and 2.4 times more likely to have undiagnosed hypertension than those who were poor. In addition, individuals with moderate income were nearly 7 times more likely to have high blood pressure compared to those who were poor. These results provide important information on how to target efforts to address hypertension in the Kuna population.

This study provides updated prevalence estimates for the island dwelling Kuna Indian population, and is one of the first studies examining the drivers of hypertension among this remote indigenous population in the past decade. Previous studies in the Kuna focused on the influences of age, migration, and diet and reported little to no hypertension among island dwelling Kuna [19–24]. Our findings showed the prevalence of hypertension among the island dwelling Kuna to be 6.2% as defined by > = 140/90 mmHg cutoff, and 16.6% as defined by > = 130/80 mmHg cutoff. Though this prevalence is lower than the overall population of Panamá estimate of 29.6% (which used a > =140/90 cutoff), it is higher than the 2.2% noted for island Kunas in studies conducted in 1997, and in a similar range of the 11.6% found in the Kuna living on the mainland of Panamá in 2011 [13, 25]. Differences in populations measured in 2011 and our sample in 2013 include this sample being island dwelling and having limited access to the mainland, whereas the prior study population included Kuna who either lived in Panama City or living in indigenous zones that are accessible to the city by land. We also found differences by sex and income for both the prevalence of hypertension and undiagnosed hypertension. Males were more likely to be hypertensive and be unaware of their hypertension, suggesting interventions should be targeted to reach this demographic. Individuals who were poor had lower prevalence of hypertension and undiagnosed hypertension than higher income groups. This observation is interesting given a similar study of an indigenous population in central Brazil found neither sex nor family income to be associated with hypertension [15].

These novel observations will require more investigation to develop targeted interventions that address chronic disease recognition and treatment in the Kuna. To properly design interventions, it will be helpful to gather information on how increasing income is associated with an increased prevalence of hypertension in a remote and overall low to moderate income population. Most studies find lower income groups are at higher risk of disease, so a better understanding of the drivers for this relationship will be important in developing a culturally appropriate intervention [29]. In addition, given these results, work is needed to address undiagnosed hypertension in this population. Studies in indigenous populations have found high levels of undiagnosed hypertension, with one study reporting 64% of Western Alaska Natives unaware they had hypertension, and a second study finding 85% of a Fulani tribe in Africa being unaware of hypertension [14, 30]. Understanding the root cause and the demographics most influential in accessing care will be essential in decreasing undiagnosed hypertension and improving the health outcomes for the Kuna.

This study is strengthened by Indigenous Health International’s long-standing relationship with the Kuna and the participatory nature of the data collection. This continuing relationship will allow for further studies and effective utilization of the results. However, this study does have imitations. First, prevalence was collected during a single time point and therefore cannot measure change overtime or comment on causation between sociodemographic factors and hypertension. Second, this was a relatively small sample size and can be further strengthened with a larger sample group, particularly if taken from additional islands. Lastly, as is true in many indigenous studies, the generalizability of these findings is limited to the Kuna population and cannot be assumed to apply to other indigenous populations. However, this study provides a strong template which can be used to investigate other indigenous populations.

## Conclusion

In conclusion, the prevalence of hypertension in the Kuna and frequency of undiagnosed hypertension has unique sociodemographic drivers, the most consistent being income. We found significantly higher and more frequently undiagnosed hypertension in males, older age, and higher income. Investigating the impact of these influences and better understanding the underlying drivers remains vitally important in helping to improve the health of the Kuna Indians and other vulnerable indigenous populations.

## Data Availability

The dataset generated and analyzed during the current study is not publicly available because of confidentiality agreement with the study communities. Data are however available from the authors upon reasonable request and with permission of the leadership of the study communities. Requests can be initiated by contacted the corresponding author.

## Abbreviations

CI: Confidence Interval
CVD: Cardiovascular disease
IHI: Indigenous Health International
OR: Odds Ratio
SD: Standard Deviation
US: United States
WIRB: Western Institutional Review Board
